# Alerts in Clinical Decision Support Systems (CDSS): A Bibliometric Review and Content Analysis

**DOI:** 10.3390/healthcare10040601

**Published:** 2022-03-23

**Authors:** Shuo-Chen Chien, Ya-Lin Chen, Chia-Hui Chien, Yen-Po Chin, Chang Ho Yoon, Chun-You Chen, Hsuan-Chia Yang, Yu-Chuan (Jack) Li

**Affiliations:** 1 Graduate Institute of Biomedical Informatics, College of Medical Science and Technology, Taipei Medical University, Taipei 110, Taiwan; alanjian@gmail.com (S.-C.C.); end1859612@gmail.com (Y.-L.C.); sophie123@tmu.edu.tw (C.-H.C.); drharvey9846@gmail.com (Y.-P.C.); jimchen@w.tmu.edu.tw (C.-Y.C.); lovejog@tmu.edu.tw (H.-C.Y.); 2 International Center for Health Information and Technology, College of Medical science and Technology, Taipei Medical University, Taipei 110, Taiwan; 3 Office of Public Affairs, Taipei Medical University, Taipei 110, Taiwan; 4 Department of Medicine, Brigham and Women’s Hospital and Harvard Medical School, Boston, MA 02115, USA; 5 Big Data Institute, University of Oxford, Oxford OX3 7LF, UK; changho.yoon@gmail.com; 6 Nuffield Department of Population Health, University of Oxford, Oxford OX3 7LF, UK; 7 Department of Radiation Oncology, Taipei Municipal Wan Fang Hospital, Taipei 110, Taiwan; 8 Information Technology Office in Taipei Municipal Wan Fang Hospital, Taipei Medical University, Taipei 110, Taiwan; 9 Department of Dermatology, Taipei Municipal Wan Fang Hospital, Taipei 110, Taiwan

**Keywords:** decision support systems, clinical, medical order entry systems, alert fatigue, health personnel, bibliometrics, review literature as topic

## Abstract

A clinical decision support system (CDSS) informs or generates medical recommendations for healthcare practitioners. An alert is the most common way for a CDSS to interact with practitioners. Research about alerts in CDSS has proliferated over the past ten years. The research trend is ongoing with new emerging terms and focus. Bibliometric analysis is ideal for researchers to understand the research trend and future directions. Influential articles, institutes, countries, authors, and commonly used keywords were analyzed to grasp a comprehensive view on our topic, alerts in CDSS. Articles published between 2011 and 2021 were extracted from the Web of Science database. There were 728 articles included for bibliometric analysis, among which 24 papers were selected for content analysis. Our analysis shows that the research direction has shifted from patient safety to system utility, implying the importance of alert usability to be clinically impactful. Finally, we conclude with future research directions such as the optimization of alert mechanisms and comprehensiveness to enhance alert appropriateness and to reduce alert fatigue.

## 1. Introduction

Making mistakes is human; even the experts are not exempt [1]. In order to prevent mistakes, alerts, defined as notifications or warnings that highlight the risk of danger, have been widely utilized in many fields [2,3,4]. This includes healthcare, where clinical decision support systems (CDSS) commonly use alerts to notify clinicians of actual or potential errors [5,6]. Previous studies have confirmed that alerts are an efficient way to prevent medication errors and streamline clinical workflow [7,8,9].

A CDSS is a computer program based on evidence-based clinical guidelines with or without artificial intelligence (AI), designed to support healthcare providers in identifying problems, resolving them, and reducing errors [10,11,12,13]. Mathematical models of AI are the main component of a CDSS and have been extensively studied [14,15,16]. Furthermore, the AI-based CDSS, used to help against the COVID-19 pandemic, has been widely used in many previous studies [17,18,19]. An example of a CDSS is the computerized physician order entry system (CPOE), which provides physicians with functions such as medication prescription, and laboratory and radiology orders [20,21,22,23,24]. There are different ways for alerts to appear in a CPOE, e.g., pop-up windows, interruptive vs. non-interruptive, active vs. passive alerts [25]. Moreover, alerts are not necessarily clinically relevant; administrative alerts too may frequently feature [26]

Bibliometric analysis is one method of synthesizing a literature review through citation statistics, which could help depict trending concepts in the field of interest and has been used in CDSS applications [27]. For example, Olufisayo Olusegun (2021) systematically reviewed the publications related to the appropriateness of CDSS alerts from 1997 to 2018 [28]. Cemal Aktürk (2021) used the bibliometric method to explore the concept of CDSS conducted between 2016 and 2021 [29]. However, the study of bibliometric analysis to uncover CDSS-alert-related articles is lacking. Therefore, different perspectives provided to this field are needed.

In this study, we aimed to use bibliometric and content analysis methods to explore the trend in using CDSS alerts in the last ten years. Especially, we included the implementation, evaluation, and optimization of CDSS. Along with the knowledge structure derived from bibliometric analysis, we conclude with a broad assessment of current CDSS research as well as suggestions for future research directions.

## 2. Materials and Methods

Literature review methods include systematic literature review, meta-analysis, bibliometric analysis, and content analysis [30]. We first extracted the bibliographic data published between 2011 and 2021 in the Web of Science (WoS) database. Subsequently, bibliometric and content analysis (both quantitative and qualitative types) were combined to explore our research question.

### 2.1. Bibliometric Analysis

Bibliometric analysis is a quantitative method using the bibliographic information of publications to analyze their impacts and relationships [27,31,32]. The well-known bibliometric data include authorships, citations, references, and keywords. The following four approaches using different bibliometric data (e.g., keywords, citations, and authorships) were used in this study to capture the conceptual, intellectual, and social networks [27].

#### 2.1.1. Performance Analysis Using Citation Numbers

To determine the contribution of an article, we used the citation number as how many times an article is cited by others to represent its influence on science [27]. There are other ways to express the impact of a scientific article; however, a citation number is an objective and repeatable index that reflects an article’s relationship and the degree of relevance with others [31]. It is hypothesized that researchers refer to an article when it is regarded as relevant and of good quality. Specifically, total global citations (TGC) denotes the number of citations in the entire WoS database, whereas total local citations (TLC) denotes the number of citations among the included articles based on our search criteria.

#### 2.1.2. Citation Analysis

Citation analysis is the most commonly used approach in bibliometrics as it depicts the intellectual structure of the research field [32]. One method in citation analysis is bibliometric coupling. This method relates articles by comparing the citations of two papers and determining the similarity. Precisely, bibliographic coupling requires at least two publications to cite the same article in their references [33,34].

#### 2.1.3. Trending Research Concepts Using Keywords

Core keywords represent the conceptual structure of the specific field [35]. We used “KeyWords Plus” (provided by the WoS databases) to reveal the emerging keywords of our research question [36].

#### 2.1.4. Country Collaboration Map

A social structure of a scientific research field can be well represented by delineating the networks between countries [27]. Country collaboration is established when two countries appear in the same article as the origins of the affiliations of authors. A country collaboration map was therefore generated to visualize the geographical relationships of our research question.

### 2.2. Content Analysis

Content analysis is a kind of qualitative study that is defined as “a research method for the subjective interpretation of the content of text data through the systematic classification process of coding and identifying themes and patterns” [37]. A valid process of theme coding is often required to evaluate the content of included articles [38]. With the completion of coding, researchers summarize and interpret the coding concepts.

### 2.3. Data Extraction Process

On the basis of the Preferred Reporting Items for Systematic Reviews and Meta-Analyses (PRISMA) guidelines, there were two stages in the data extraction process [39]. First, the bibliographic data were collected from Clarivate Analytics’ Web of Science (WoS), which encompasses 12,000 ISI-indexed journals [40]. The keyword search was conducted in August 2021. We considered a combination of keywords from (1) (“alert*” OR “alarm*” OR “warning*” OR “reminder*” OR “notification”) and (2) (“CPOE” OR “CDS” OR “computerized physician order entry” OR “computerized provider order entry” OR “clinical decision support”). Proceedings papers and early access papers were included in our study. Papers published before 2011 were excluded. Additionally, we excluded studies published as reviews, editorials, letters, books, corrections, and items. Non-English articles were also excluded.

In the second stage, a detailed examination of the articles was applied through reading titles and abstracts. We removed articles with the terms “cds” and “cpoe” related to other meanings; for example, cds was found to be related to Calgary Depression Scale, a clinical scale for patient-reported outcomes. Studies that investigated the alarms of medical devices, such as electroencephalography (EEG), electrocardiography (ECG), infusion pumps, and ventilators, were also excluded. Furthermore, publications that focused on the innovation and design of algorithms that alter the functions of CDSS were not considered as the scope of this study is to evaluate the application of CDSS alerts.

Lastly, we selected a list of crucial articles based on TGC and TLC for content analysis. We included articles with more than or equal to 40 TGC and 10 TLC. The coding process was conducted through the thorough reading of these articles, and thus a concept matrix was developed with a list of primary information, such as study design, alert types, and alert topics [41]. Our coding categories followed the suggestions from Gaur and Kumar (2018) [38].

We used the R package Bibliometrix and Matplotlib package v3.3.4 using Python 3.8.8 for bibliographic analysis and visualization [42,43].

## 3. Results

### 3.1. Initial Paper Selection Result

**Figure 1:**
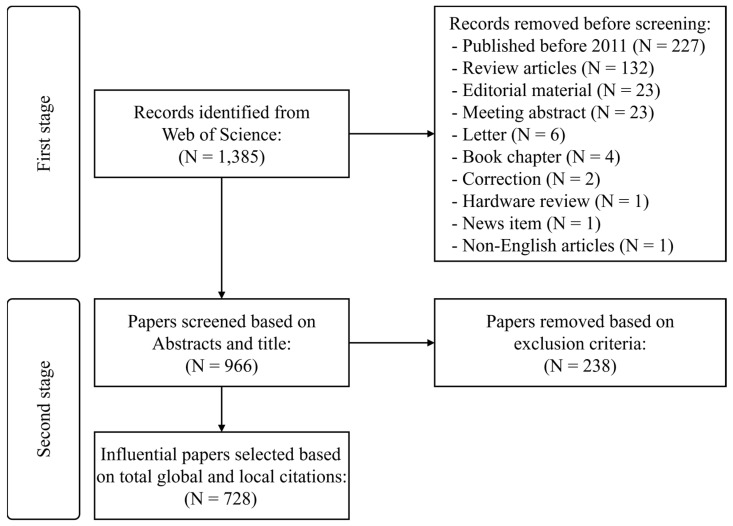
Paper selection process.

The WoS database returned 1385 records based on the keywords search (Figure 1). In the first stage, we removed papers published before 2011 (N = 227), review articles (N = 132), editorial materials (N = 23), meeting abstracts (N = 23), letters (N = 6), books (N = 4), corrections (N = 2), and news items (N = 1); we additionally removed one paper for being non-English. Subsequently, in the second stage, we screened the titles and abstracts of the remaining 966 articles and excluded 238 papers due to unsuitable content. Finally, a cohort of 728 papers was included for bibliometric analysis. The distribution of yearly publications is presented in Figure S1.

### 3.2. Most Relevant Journals, Authors, Institutions, and Articles

Performance analysis using citation numbers produced lists of the most relevant journals, articles, institutions, and authors. A total of 728 articles were published by 211 journals. Most of the top-ranked journals belonged to the categories of medical informatics, clinical informatics, and bioinformatics (Table 1). Journal of the American Medical Informatics Association (N = 93, 22.7%), Applied Clinical Informatics (N = 74, 18.1%), and International Journal of Medical Informatics (N = 47, 11.5%) were the top three productive journals, all of which are categorized into the field of medical informatics by the Science Citation Index Expanded (SCIE). PLoS One, a multidisciplinary journal, was also listed in the top 10. Notably, journals categorized in fields other than medical informatics by the SCIE, such as American Journal of Health-System Pharmacy (SCIE category: Pharmacology and Pharmacy) and the Journal of General Internal Medicine (SCIE category: Health Care Sciences & Services), were also listed, implying the emerging importance of informatics application in subfields of medicine.

**Figure 2:**
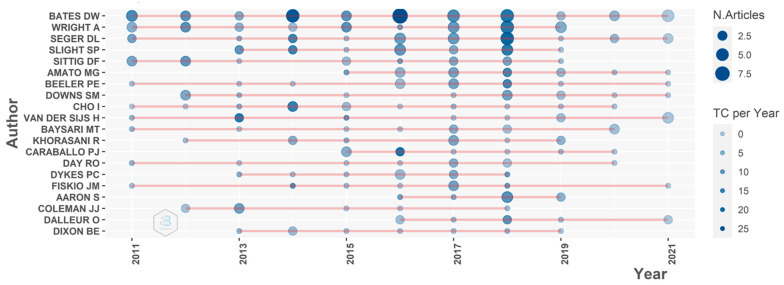
Top 20 authors’ publication productivity time.

Bates D.W., Wright A., Seger D.L., and Slight S.P. were the authors with the greatest productivity with high impact over the past ten years (Figure 2). These four researchers focus on the fields of CDSS and patient/medication safety. Notably, all four authors were affiliated with Brigham and Women’s Hospital in the included studies. In terms of the overall author’s impact in the previous ten years, Bates D.W. was the most influential researcher (TGC = 1031), followed by Wright A. (TGC = 583), Seger D.L. (TGC = 542), and Slight S.P. (TGC = 462).

The top three most influential institutions were the University of Washington (N = 86), Brigham and Women’s Hospital (N = 79), and Harvard Medical School (N = 70) (Table 2). Taipei Medical University (N = 47) was the only institution not located in the United States and ranked eighth in the list.

Regarding the impact of individual studies, the top 20 most relevant articles had TLCs greater than 15 and TGCs greater than 31 (Table S1). The most impactful paper in our research is Nanji et al. (2014) [44]. Eight out of the top 20 most relevant articles studied the effect of medication-related alerts in CPOE, particularly drug–drug interaction reminders.

### 3.3. Bibliographic Coupling

Bibliographic coupling portrays the scientific network between publications. The nodes and edges represent the coupled articles and the associations, respectively (Figure S2). Most articles could be classified into either Class A or Class B, where Class A was related to the implementation of medication-related alerts, and Class B focused on alert optimization to deliver the best practice.

### 3.4. Trending Research Concepts Using Keywords

**Figure 3:**
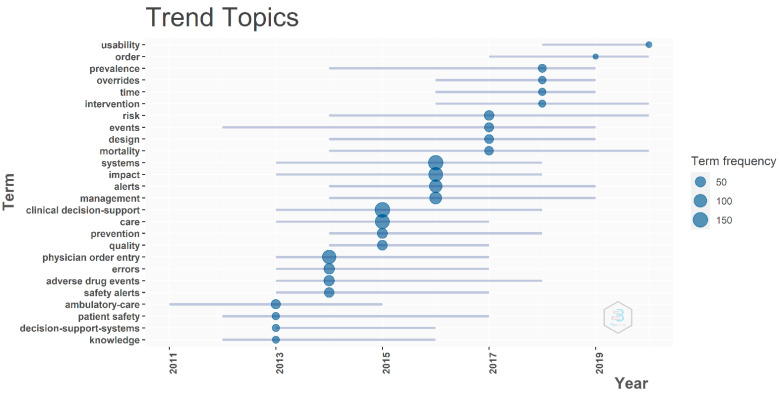
Core keywords analysis for trending topics.

In an attempt to grasp the gradual change of research concepts, first, the medium number of occurrences of each keyword (KeyWords Plus) was calculated. These numbers were then compared, and the top 4 frequent words of each year were represented in dots (Figure 3). Overall, the most frequent terms were “clinical decision-support” (N = 152), “systems” (N = 147), “care” (N = 136), and “impact” (N = 130), followed by “physician order entry” (N = 122), “alerts” (N = 98), “errors” (N = 53), and “adverse drug events” (N = 51). The emerging terms over the last three years included “usability” (N = 11) and “overrides” (N = 19). This finding corresponds to our research question in which the trend of CDSS alert research has transferred from system design and the reduction of medical errors with the aid of CDSS to the usability and override issues.

### 3.5. Country Collaboration Map

**Figure 4:**
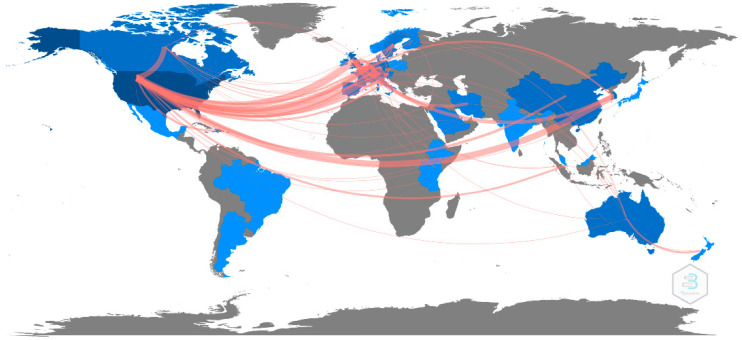
The country collaboration map for CDSS alert studies.

Figure 4 shows the country relationship of the included publications. The United States was the most active country in CDSS alert research and demonstrated the strongest association with the United Kingdom (N = 19), followed by South Korea (N = 15) and Canada (N = 12). In addition, unlike countries from other continents, Asian countries tended to work with countries from other continents rather than collaborate with each other.

### 3.6. Content Analysis

The twenty-four most impactful studies (TGC ≥ 40 and TLC ≥ 10) were extracted from the study cohort of 728 articles (Table S2) to conduct the content analysis. The majority of these studies were observational (83.3%) or used qualitative methods (87.5%) such as focus groups and interviews. Only four studies were interventional, namely, involving the modification of CPOE alert systems. Most studies were conducted in hospitals (70.8%), and only one study was done in community settings. The study populations were most commonly composed of physicians, followed by nurses and other personnel. Regarding the issues of interest in CDSS research, most studies concentrated on medication problems, such as adverse drug events (ADE) or drug–drug interactions (DDI), using alerts of interruptive or hard-stop design. This result specifically answers our research question about the research mainstreams of CDSS alerts. Finally, based on our results, we summarized the research gaps of current studies in the CDSS alert system and provided corresponding suggestions, as in Table 3.

## 4. Discussion

In this study, we used bibliometric and content analysis methods to explore the concepts of alerts in CDSS. Given the fast-growing body of research regarding alerts in CPOE and CDSS, a bibliometric analysis served as a timely summary of this technology’s recent historical trends and current focuses [29]. Indeed, the importance of a CDSS has led to a growing trend of research in the field of biomedical informatics [45]. In addition, it has recently shown its robust ability to assist people during the coronavirus pandemic period [46,47,48,49].

Keyword analysis helped to provide an insight into the evolution of hot topics over the past ten years in the field of CDSS alerts. In the earlier years, most of this research focused on the design and development of the CDSS (and its alerts), patient safety, and the reduction of adverse drug events [50,51]; recently, the focus has changed to the evaluation of the CDSS alert efficiency and usability [52]. Since the number of alerts used in CDSS has increased, studies regarding alert performance have become more popular. Common metrics to estimate the alert performance and describe the alert fatigue phenomenon include alert override rate and alert dwell time [53,54]. How to reduce the total number of alerts, increase the alert acceptance, and trigger the alert precisely have become hot research topics in this theme.

Content analysis was used to obtain a comprehensive understanding of influential publications in our study cohort, of which 24 items met the criteria (TGC ≥ 40 and TLC ≥ 10). Most healthcare providers used the hard-stop or interruptive alerts as displayed in the process [55,56]. Compared to the soft-stop or passive alerts, they have been proven to be a sufficient way to prevent medical errors [25]. However, the majority of alerting system designs are rule-based/silo nowadays, which means the alerts cannot be triggered depending on the different situations [57]. Thus, alert implementation will not increase the clinical decision value equivalently [58,59]. A context-aware solution based on machine learning should be conducted in future studies [60,61].

We also labeled the study location, population, and alert topics for the content analysis. The result shows that the receivers of CDSS alerts are not restricted to physicians but also comprise other medical personnel from various clinical settings [50,62]. These professional groups execute different clinical processes, influencing their importance judgment on the same alerts. Therefore, it is essential to explore the perspective discrepancy among these characteristics. Most of the studies focused on the clinical alerts (e.g., drug–drug interaction, adverse drug events, and allergy), which reached varying degrees of success in improving patient safety [63,64]. However, both administrative (low or no clinical relevance) and clinical alerts exist in the CDSS. Only focusing on specific ones may limit the improvements to the alert system [26]. We suggest that a comprehensive analysis for all types of CDSS alerts should be considered when designing a similar study in the future.

Here, we used the bibliometric methods to demonstrate the research landscape, including studies about implementation, evaluation, and optimization of CDSS alerts in the past ten years. Therefore, based on the results, we have the following suggestions while re-designing or conducting the study related to CDS alert systems. First, researchers should adopt multiple metrics to comprehensively collect a breadth of perspectives when assessing the effectiveness of alerting systems. Second, CDS system designers may consider implementing the AI-based precision alert system in the next generation of CDSS. Lastly, all types of CDSS alerts should be included in the study to grasp a holistic view of alert use in the settings. By following these guidelines, the clinical workflows may be improved, increasing alert efficiency with conceivable benefits to patient safety [65].

### Limitations

Our study has several limitations. First, we only adopted the WoS as the source of bibliographic data. Not including publications in other databases such as Scopus and PubMed may have limited our findings. Thus, a comprehensive analysis will be conducted in our future study. Second, we only analyzed the studies in the last ten years, while the theme has been developed for over 30 years. However, it is more convincing to depict the trend topics based on the latest information. Lastly, we only included English-written publications in our analysis.

## 5. Conclusions

Our study depicted a comprehensive overview of the field of CDSS alerts. We found over 700 publications in the last ten years. The results demonstrated the trend of CDSS alert research with several aspects, including the contribution of journals, authors, institutions, and countries, keyword analysis, and content analysis. The findings of this study showed that research mainly focused on improving the quality of the CDSS alert system and increasing alert efficiency. We also provided some future directions for the research in this topic, encouraging researchers to design or validate an alert system towards the goal of decreasing alert fatigue and improving patient safety.

## Figures and Tables

**Table 1:** Most influential journals (sorted by the number of publications).

#	Journals	Item		TGC	TGC per Item	IF (2020)
		N	%			
1	Journal of the American Medical Informatics Association	93	22.7	2798	30.1	4.50
2	Applied Clinical Informatics	74	18.1	232	3.1	2.34
3	International Journal of Medical Informatics	47	11.5	558	11.9	4.05
4	BMC Medical Informatics and Decision Making	34	8.3	258	7.6	2.80
5	American Journal of Health-system Pharmacy	16	3.9	289	18.1	2.64
6	JMIR Medical Informatics	16	3.9	18	1.1	2.96
7	PLoS ONE	15	3.7	157	10.5	3.24
8	International Journal of Clinical Pharmacy	14	3.4	57	4.1	2.05
9	Journal of Clinical Pharmacy and Therapeutics	11	2.7	89	8.1	2.51
10	Drug Safety	9	2.2	201	22.3	5.61
11	BMJ Quality & Safety	9	2.2	88	9.8	7.04
12	Artificial Intelligence in Medicine	9	2.2	63	7.0	5.33
13	CIN-COMPUTERS INFORMATICS NURSING	9	2.2	32	3.6	1.99
14	Journal of General Internal Medicine	8	2.0	311	38.9	5.13
15	Journal of Biomedical Informatics	8	2.0	265	33.1	6.32
16	Pharmacoepidemiology and Drug Safety	8	2.0	135	16.9	2.89
17	Journal of Medical Systems	8	2.0	40	5.0	4.46
18	Medical Care	7	1.7	141	20.1	2.98
19	American Journal of Medical Quality	7	1.7	36	5.1	1.85
20	American Journal of Clinical Pathology	7	1.7	34	4.9	2.49

Abbreviations: N = Number of publications, TGC = Total global citations, IF = Impact factor.

**Table 2:** Most influential institutions (sorted by the number of publications).

#	Institutions	N	Location
1	University of Washington	86	Seattle, WA, USA
2	Brigham and Women’s Hospital	79	Boston, MA, USA
3	Harvard Medical School	70	Boston, MA, USA
4	University of Pittsburgh	68	Pittsburgh, PA, USA
5	Harvard University	66	Boston, MA, USA
6	Vanderbilt University	65	Nashville, TN, USA
7	Stanford University	49	Stanford, CA, USA
8	Taipei Medical University	47	Taipei, TW
9	Mayo Clinic	43	Scottsdale, AZ, USA
10	University of Pennsylvania	43	Philadelphia, PA, USA
11	Columbia University	36	New York, NY, USA
12	Partners HealthCare International	33	Boston, MA, USA
13	Indiana University School of Medicine	32	Indianapolis, IN, USA
14	Cincinnati Children’s Hospital Medical Center	31	Cincinnati, OH, USA
15	University of Michigan	31	Ann Arbor, MI, USA
16	Case Western Reserve University	28	Cleveland, OH, USA
17	University of California, Los Angeles	28	Los Angeles, CA, USA
18	Icahn School of Medicine at Mount Sinai	27	New York, NY, USA
19	Indiana University School of Medicine	26	Indianapolis, IN, USA
20	Oregon Health & Science University	26	Portland, OR, USA

Abbreviations: N = Number of publications.

**Table 3:** The summary of current research gaps and suggestions.

#	Current Research Gap	Suggestion
1	Usually used only a single metric to evaluate the alert system’s efficiency.	Adopting multiple metrics to comprehensively collect perspectives.
2	Most of the studies focused on specific types of CDSS alerts.	Consider including all types of CDSS alerts to grasp a holistic view of alert usage.
3	The majority of alerting system designs are rule-based/silo.	An AI-based precision alert system should be considered to implement in the next generation of CDSS.

## Data Availability

The data presented in this study are available in this manuscript.

## Supplementary Materials

The following supporting information can be downloaded at: https://www.mdpi.com/article/10.3390/healthcare10040601/s1, Figure S1. Distribution of yearly publications and the yearly averages of TGC per article, Figure S2. Bibliographic coupling (A = medication-related cluster and B = best practice cluster), Table S1. Ranking of top 20 articles (sorted by TLC), Table S2. The content analysis for 24 most impactful articles (TGC ≥ 40 and TLC ≥ 10). References [44,50,51,55,56,58,62,66,67,68,69,70,71,72,73,74,75,76,77,78,79,80,81,82,83,84,85] are cited in the supplementary materials.
