# Supplementary material for: Alerts in Clinical Decision Support Systems (CDSS): A Bibliometric Review and Content Analysis
Source: Healthcare (Basel). 2022 Mar 23;10(4):601. doi: 10.3390/healthcare10040601 (PMC9028311; doi:10.3390/healthcare10040601)
Supplement: Supplementary file 1 [file healthcare-10-00601-s001.zip › healthcare-1647493-supplementary.pdf]

Table S1. Ranking of top 20 articles (sorted by TLC)

#	Article	Journal	TLC	TGC	TLC/TGC Ratio (%)
**1**	Nanji KC et al. (2014) [1]	J AM MED INFORM ASSN	51	116	44.0
**2**	Phansalkar S et al. (2013) [2]	J AM MED INFORM ASSN	40	112	35.7
**3**	Payne TH et al. (2015) [3]	J AM MED INFORM ASSN	38	85	44.7
**4**	Seidling HM et al. (2011) [4]	J AM MED INFORM ASSN	34	77	44.2
**5**	McCoy AB et al. (2012) [5]	J AM MED INFORM ASSN	31	70	44.3
**6**	Bryant AD et al. (2014) [6]	APPL CLIN INFORM	29	92	31.5
**7**	Ancker JS et al. (2017) [7]	BMC MED INFORM DECIS	29	127	22.8
**8**	Seidling HM et al. (2014) [8]	INT J MED INFORM	25	50	50.0
**9**	Coleman JJ et al. (2013) [9]	BMC MED INFORM DECIS	24	58	41.4
**10**	Riedmann D et al. (2011) [10]	BMC MED INFORM DECIS	23	44	52.3
**11**	Slight SP et al. (2013) [11]	PLOS ONE	22	51	43.1
**12**	Nanji KC et al. (2018) [12]	J AM MED INFORM ASSN	20	48	41.7
**13**	Saverno KR et al. (2011) [13]	J AM MED INFORM ASSN	18	79	22.8
**14**	Carspecken W et al. (2013) [14]	PEDIATRICS	18	59	30.5
**15**	Bell GC et al. (2014) [15]	J AM MED INFORM ASSN	17	122	13.9
**16**	Wright A et al. (2016) [16]	J AM MED INFORM ASSN	17	56	30.4
**17**	Russ AL et al. (2012) [17]	INT J MED INFORM	16	55	29.1
**18**	Eppenga WL et al. (2012) [18]	J AM MED INFORM ASSN	16	31	51.6
**19**	Scott GPT et al. (2011) [19]	J AM MED INFORM ASSN	15	36	41.7
**20**	Duke JD et al. (2013) [2]	J AM MED INFORM ASSN	15	33	45.6

Abbreviations: TLC = Total local citations received, TGC = Total global citations received, J AM MED INFORM ASSN = Journal of the American Medical Informatics Association, BMC MED INFORM DECIS = BMC Medical Informatics and Decision Making, INT J MED INFORM = International Journal of Medical Informatics.

Table S2. The content analysis for 24 most impactful articles (TGC ≥ 40 & TLC ≥ 10)

Articles	TLC	TGC	TLC/TGC Ratio	Study type	Study location	Study population	Alerts Type	Alert Topic	Analysis method
(Nanji KC et al., 2014) [7]	51	116	0.44	Observational	Outpatient	Physician/Nurse/Other clinician	Not specified	ADE/Other recommendation	Quali-Quanti
(Phansalkar S et al., 2013) [2]	40	112	0.36	Observational	Not specified	NA	Soft-stop	DDI	Quali-Quanti
(Payne TH et al., 2015) [3]	38	85	0.45	Observational	N/A	Physician/IT/Academia /Diverse backgrounds expert	Not specified	DDI	Qualitative
(Seidling HM et al., 2011) [4]	34	77	0.44	Observational	Outpatient/Inpatient	Not specified	Not specified	DDI	Quantitative
(McCoy AB et al., 2012) [5]	31	70	0.44	Focus group/Observational	Inpatient	Clinician	Not specified	ADE/Laboratory/Other recommendation	Quali-Quanti
(Ancker JS et al., 2017) [7]	29	127	0.23	Observational	Outpatient	Physician/Nurse	Interruptive	Drug/Clinical practice	Quantitative
(Bryant AD et al., 2014) [6]	29	92	0.32	Observational	Inpatient	Physicians	Interruptive	ADE	Quantitative
(Seidling HM et al., 2014) [8]	25	50	0.50	Observational	Outpatient/Inpatient	N/A	Not specified	DDI	Quali-Quanti
(Coleman JJ et al., 2013) [9]	24	58	0.41	Focus group	Not specified	Pharmacist	N/A	Allergy/DDI	Qualitative

(Riedmann D et al., 2011) [10]	23	44	0.52	Focus group	Not specified	CPOE expert	N/A	ADE	Qualitative
(Slight SP et al., 2013) [11]	22	51	0.43	Observational	Outpatient	Physician	Hard-stop	DDI	Quali-Quanti
(Nanji KC et al., 2018) [12]	20	48	0.42	Observational	Inpatient	Physician/ Nurse/Other clinician	Hard-stop	ADE/ Substitution/Other recommendation	Quali-Quanti
(Saverno KR et al., 2011) [13]	18	79	0.23	Interventional / On-site visit	Community/Inpatient/ Other	Pharmacist	Hard-stop	DDI	Quali-Quanti
(Carspecken CW et al., 2013) [14]	18	59	0.31	Observational	PICU	One specific patient	Pop-up	Allergy	Qualitative
(Bell GC et al., 2014) [15]	17	122	0.14	Interventional	Inpatients/ Outpatient/Home	Physician/ Pharmacist	Pop-up/Email	Allergy	Quali-Quanti
(Wright A et al., 2016) [16]	17	56	0.30	Observational	Entire hospital	Chief Medical Information Officers	Not mentioned	All types of EHR alert	Quali-Quanti
(Russ AL et al., 2012) [17]	16	55	0.29	Observations/ Interview	Outpatient	Physician/ Nurse/ Pharmacist	Pop-up	ADE	Qualitative
(Scheife RT et al., 2015) [20]	12	66	0.18	Focus group	N/A	Diverse backgrounds expert	N/A	DDI	Qualitative
(Russ AL et al., 2014) [21]	12	50	0.24	Interventional	N/A	Prescriber	Not mentioned	ADE	Qualitative
(Topaz M et al., 2016) [22]	12	45	0.27	Observational	Inpatient	Provider	Hard-stop	Allergy	Quali-Quanti

(Romano MJ et al., 2011) [23]	11	192	0.06	Observational	Outpatient/ Emergency	Physician	N/A	N/A	Qualitative
(Baysari MT et al., 2011) [24]	11	46	0.24	Observational	All wards(Except ED and ICU)	Physician	Hard-stop	All types of EHR alert	Quali-Quanti
(Wright A et al., 2011) [25]	11	45	0.24	Observational	N/A	N/A	N/A	N/A	Qualitative
(Hoffman JM et al., 2014) [26]	10	143	0.07	Interventional	All primary clinical services	Physician	Interruptive	Allergy	Qualitative

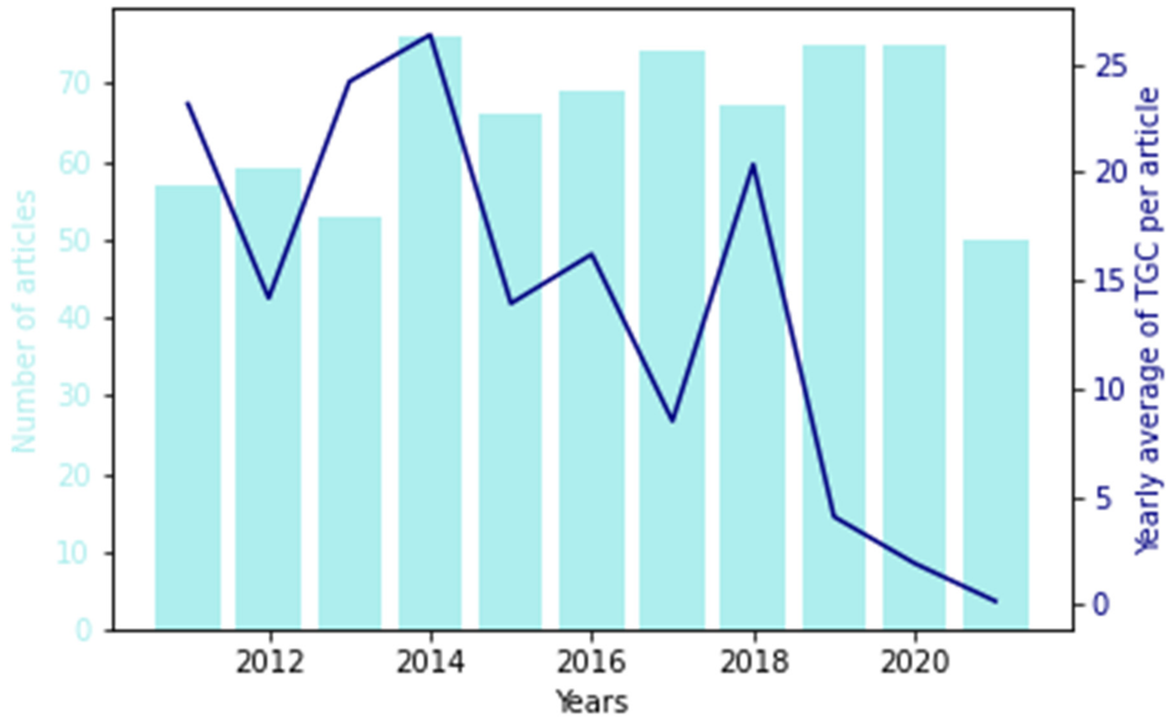

Figure S1. Distribution of yearly publications and the yearly averages of TGC per article

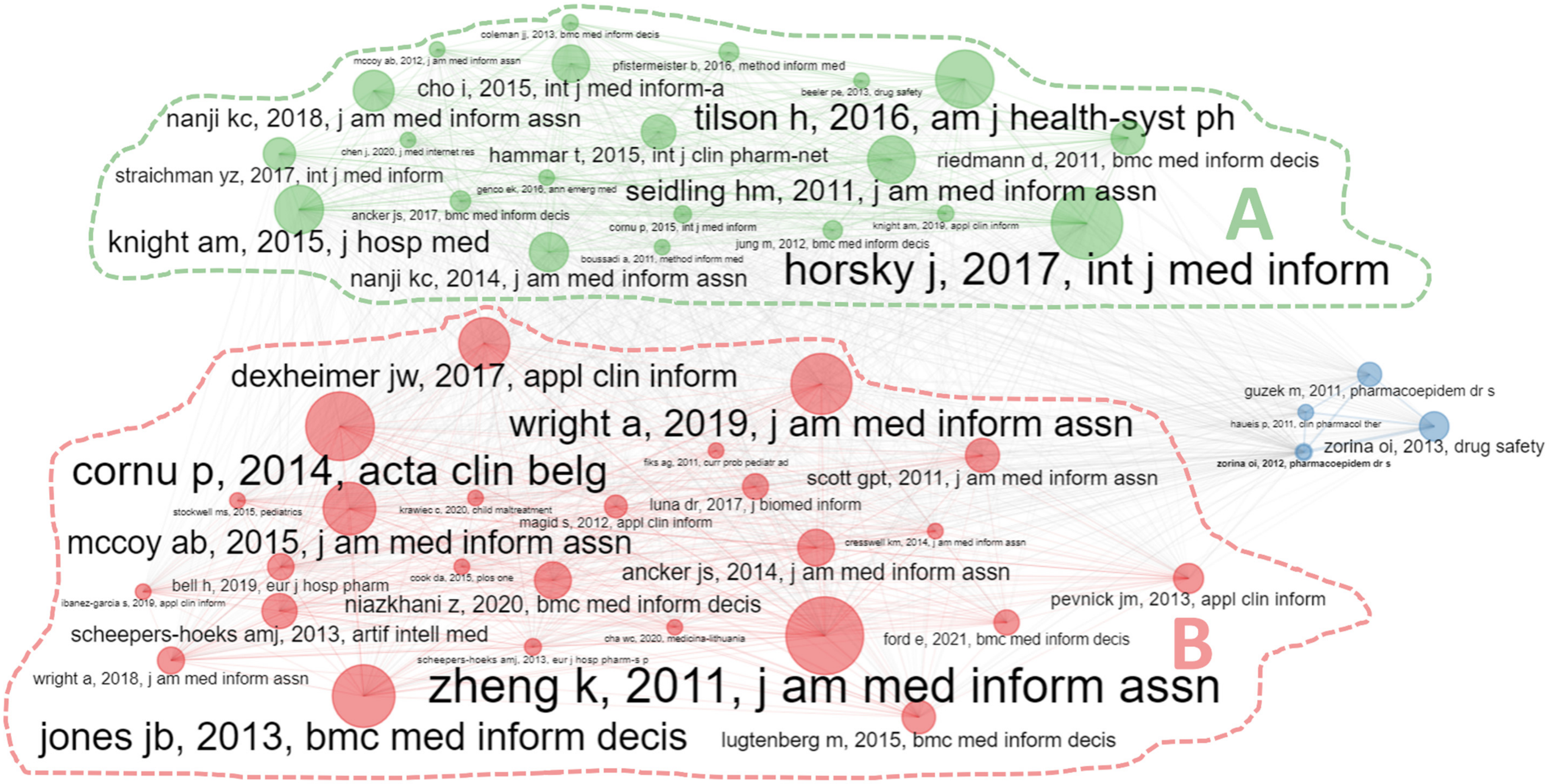

Figure S2. Bibliographic coupling (A = Medication-related cluster, B = Best practice cluster)

1. Nanji, K.C., et al., *Overrides of medication-related clinical decision support alerts in outpatients.* 2014. **21**(3): p. 487-491.
2. Phansalkar, S., et al., *Drug—drug interactions that should be non-interruptive in order to reduce alert fatigue in electronic health records.* 2013. **20**(3): p. 489-493.
3. Payne, T.H., et al., *Recommendations to improve the usability of drug-drug interaction clinical decision support alerts.* 2015. **22**(6): p. 1243-1250.
4. Seidling, H.M., et al., *Factors influencing alert acceptance: a novel approach for predicting the success of clinical decision support.* 2011. **18**(4): p. 479-484.
5. McCoy, A.B., et al., *A framework for evaluating the appropriateness of clinical decision support alerts and responses.* 2012. **19**(3): p. 346-352.
6. Bryant, A., G. Fletcher, and T.J.A.c.i. Payne, *Drug interaction alert override rates in the Meaningful Use era.* 2014. **5**(03): p. 802-813.
7. Ancker, J.S., et al., *Effects of workload, work complexity, and repeated alerts on alert fatigue in a clinical decision support system.* 2017. **17**(1): p. 1-9.
8. Seidling, H.M., et al., *What, if all alerts were specific–estimating the potential impact on drug interaction alert burden.* 2014. **83**(4): p. 285-291.
9. Coleman, J.J., et al., *On the alert: future priorities for alerts in clinical decision support for computerized physician order entry identified from a European workshop.* 2013. **13**(1): p. 1-8.
10. Riedmann, D., et al., *Development of a context model to prioritize drug safety alerts in CPOE systems.* 2011. **11**(1): p. 1-11.
11. Slight, S.P., et al., *Are we heeding the warning signs? Examining providers' overrides of computerized drug-drug interaction alerts in primary care.* 2013. **8**(12): p. e85071.
12. Nanji, K.C., et al., *Medication-related clinical decision support alert overrides in inpatients.* 2018. **25**(5): p. 476-481.
13. Saverno, K.R., et al., *Ability of pharmacy clinical decision-support software to alert users about clinically important drug—drug interactions.* 2011. **18**(1): p. 32-37.
14. Carspecken, C.W., et al., *A clinical case of electronic health record drug alert fatigue: consequences for patient outcome.* 2013. **131**(6): p. e1970-e1973.
15. Bell, G.C., et al., *Development and use of active clinical decision support for preemptive pharmacogenomics.* 2014. **21**(e1): p. e93-e99.
16. Wright, A., et al., *Analysis of clinical decision support system malfunctions: a case series and survey.* 2016. **23**(6): p. 1068-1076.
17. Russ, A.L., et al., *Prescribers' interactions with medication alerts at the point of prescribing: a multi-method, in situ investigation of the human–computer interaction.* 2012. **81**(4): p. 232-243.

18. Eppenga, W.L., et al., *Comparison of a basic and an advanced pharmacotherapy-related clinical decision support system in a hospital care setting in the Netherlands.* 2012. **19**(1): p. 66-71.
19. Scott, G.P., et al., *Making electronic prescribing alerts more effective: scenario-based experimental study in junior doctors.* 2011. **18**(6): p. 789-798.
20. Scheife, R.T., et al., *Consensus recommendations for systematic evaluation of drug-drug interaction evidence for clinical decision support.* Drug Saf, 2015. **38**(2): p. 197-206.
21. Russ, A.L., et al., *Applying human factors principles to alert design increases efficiency and reduces prescribing errors in a scenario-based simulation.* J Am Med Inform Assoc, 2014. **21**(e2): p. e287-96.
22. Topaz, M., et al., *Rising drug allergy alert overrides in electronic health records: an observational retrospective study of a decade of experience.* J Am Med Inform Assoc, 2016. **23**(3): p. 601-8.
23. Romano, M.J. and R.S. Stafford, *Electronic health records and clinical decision support systems: impact on national ambulatory care quality.* Arch Intern Med, 2011. **171**(10): p. 897-903.
24. Baysari, M.T., et al., *The influence of computerized decision support on prescribing during ward-rounds: are the decision-makers targeted?* J Am Med Inform Assoc, 2011. **18**(6): p. 754-9.
25. Wright, A., et al., *Governance for clinical decision support: case studies and recommended practices from leading institutions.* J Am Med Inform Assoc, 2011. **18**(2): p. 187-94.
26. Hoffman, J.M., et al., *PG4KDS: a model for the clinical implementation of pre-emptive pharmacogenetics.* Am J Med Genet C Semin Med Genet, 2014. **166c**(1): p. 45-55.
